# Microbial Modifications of Androstane and Androstene Steroids by *Penicillium vinaceum*

**DOI:** 10.3390/molecules25184226

**Published:** 2020-09-15

**Authors:** Anna Panek, Paulina Łyczko, Alina Świzdor

**Affiliations:** Department of Chemistry, Wrocław University of Environmental and Life Sciences, 50-375 Wrocław, Poland; paulina.lyczko@upwr.edu.pl (P.Ł.); alina.swizdor@upwr.edu.pl (A.Ś.)

**Keywords:** biotransformations, steroids, 19-hydroxyandrostenedione, 19-nortestololactone, lactonization, Baeyer-Villiger oxidation, *Penicillium vinaceum*

## Abstract

The biotransformation of steroid compounds is a promising, environmentally friendly route to new pharmaceuticals and hormones. One of the reaction types common in the metabolic fate of steroids is Baeyer-Villiger oxidation, which in the case of cyclic ketones, such as steroids, leads to lactones. Fungal enzymes catalyzing this reaction, Baeyer-Villiger monooxygenases (BVMOs), have been shown to possess broad substrate scope, selectivity, and catalytic performance competitive to chemical oxidation, being far more environmentally green. This study covers the biotransformation of a series of androstane steroids (epiandrosterone and androsterone) and androstene steroids (progesterone, pregnenolone, dehydroepiandrosterone, androstenedione, 19-OH-androstenedione, testosterone, and 19-nortestosterone) by the cultures of filamentous fungus *Penicillium vinaceum* AM110. The transformation was monitored by GC and the resulting products were identified on the basis of chromatographic and spectral data. The investigated fungus carries out effective Baeyer-Villiger oxidation of the substrates. Interestingly, introduction of the 19-OH group into androstenedione skeleton has significant inhibitory effect on the BVMO activity, as the 10-day transformation leaves half of the 19-OH-androstenedione unreacted. The metabolic fate of epiandrosterone and androsterone, the only 5α-saturated substrates among the investigated compounds, is more complicated. The transformation of these two substrates combined with time course monitoring revealed that each substrate is converted into three products, corresponding to oxidation at C-3 and C-17, with different time profiles and yields.

## 1. Introduction

The diversity of chemicals used by contemporary industry, medicine, and agriculture requires a constant search for new or more efficient synthetic routes and catalysts. Microbial enzymes and whole-cell catalytic systems seem to be an almost infinite reservoir of catalysts, with the benefit of relative ease of maintenance, mild reaction conditions, and low environmental track. Moreover, microbes exist in virtually all environments on Earth able to sustain life, from the polar regions [1] through dry deserts, to marine hydrothermal vents. Enzymes from such microorganisms must be able to operate in very diverse conditions of temperature (ranging from Arctic cold [2] to boiling water [3]), salinity, pH, or pressure. Although the last two decades have brought some shift in our approach to the marine, extreme, or isolated environments, from the use of natural products towards the isolation and identification of enzymes or microorganisms with desired biotransformation properties, a recent review [4] points out that the potential of catalytic activity found in these environments is still underexplored.

One of the fungal species with such underexplored potential is *Penicillium vinaceum*, first described in 1927 [5], and present in soil and marine environments. This species was shown to produce several known compounds of biochemical interest: cyclo (d-tryptophanyl-L-prolyl), citreoisocoumarin, brevianamide F, indol-3-carbaldehyde, α-cyclopiazonic acid, terretrione A [6], and one product specific to this fungus, a diketopiperazine alkaloid penicillivinacine with antimigratory activity against the human breast cancer cells similar to terretrione A [7]. On the other hand, little is known of its biotransformation potential. *P. vinaceum* was shown to be ineffective in the reduction of methyl cinnamate to 2-phenoxyethanol [8], but its enzymatic system is able to carry out effective transglycosylation leading to functionalized alkyl α-d-galactopyranosides, potentially useful biopolymers [9]. Several patent applications related to the biotransformations with *P. vinaceum* were filed: reduction of tetrahydrothiophen-3-one to (*R*)-tetrahydrothiophen-3-ol [10], conversion of eugenol to coniferyl aldehyde [11], and production of 3β-hydroxy-17a-oxa-d-homo-androst-5-en-17-one [12]. The last reaction is an example of a process constituting the main research subject of the current study: enzymatic Baeyer-Villiger oxidation of steroidal substrates.

Baeyer–Villiger oxidation (BVO), first reported in 1899 [13], involves the action of peroxides or peroxyacids on ketones, with the insertion of an oxygen atom into the carbon chain at the carbonyl group. Esters are formed from acyclic ketones, while cyclic substrates are converted to lactones. Industrial applications of BVO are diverse, ranging from the production of monomers for polymerization to obtaining antibiotic medicines, steroids, and pheromones [14]. The original oxidizers, peroxyacids, are problematic in many aspects: they are unstable or even explosive, environmentally unfriendly (each reacting peroxyacid molecule turns into a carboxylate anion which must then be disposed of), the solvents frequently contain chlorinated hydrocarbons, and the reactions are far from being optimal with regard to the substrate specificity, chemo-, regio-, or enantioselectivity. Large progress was made with the use of hydrogen peroxide as a greener, environmentally friendly oxidizer for chemical BVO [15], but even the most recent accomplishments which fine-tune chemical BVO to obtain high selectivity and yields [16] use organic solvents as reaction media. On the other hand, the use of isolated enzymes or whole-cell biocatalysts seems to be a tempting alternative able to solve the environmental issues of chemical BVO. Several recent comprehensive reviews [17,18] summarize the research into the microbial Baeyer-Villiger oxidation reactions since late 1940s, from pure research to the development of bioprocesses. The latter review concludes with such statements as “the real break-through of the BV biooxidation in industry has still not been realized. Hence, it is a prime time to seek out new targets for BV biooxidations” [18]. Interestingly, many compounds considered nowadays as biotransformation targets are steroids, the same class of substances found in the first reports on microbial Baeyer–Villiger oxidation [19,20].

Importance of steroids for life is underlined by their diverse and ubiquitous roles in cellular biochemistry: participation of cholesterol in cell membranes, digestion facilitated by bile acids, and most importantly for medicinal applications, the hormonal activity of many steroid derivatives. The last factor is crucial for the use of steroid derivatives in medicine (as anti-inflammatory and anti-cancer medications) and sport (anabolic drugs). Recent reports have turned our attention especially towards 19-hydroxy steroidal derivatives: 19-OH-androstenedione has been shown to participate in neuroendocrine trans-differentiation of prostate cancer cells [21], while a new biocatalytic route was devised [22] leading to 19-OH-cortexolone, 19-OH-androstenedione, and ultimately to numerous C19-hydroxysteroids and 19-norsteroids with antitumor and insecticidal activities. Therefore we have included representatives of 19-OH steroids in our biotransformation experiments. Biosynthetic routes from 19-nortestosterone to 19-nortestololactone were established with the use of biocatalytic fungi *Aspergillus tamarii* [23] and *Penicillium notatum* [24], but we were able to obtain in the current study better final yields than those presented in the earlier reports.

Baeyer-Villiger oxidation of steroids to steroidal lactones is one of the routes pursued in the search for new bioactive compounds. The enzymes responsible for this process are Baeyer-Villiger monooxygenases (BVMOs). While their sequences and phylogenetic tree have been intensively explored [18], much less is known about the structural aspects of BVMOs. Only recently were some bacterial and fungal BVMOs with steroids in their substrate scope crystallized and their 3D structures determined by crystallographic methods: a steroid monooxygenase from *Rhodococcus rhodochrous* [25], and a versatile polycyclic ketone monooxygenase (PockeMO) from the thermophilic fungus *Thermothelomyces thermophile* [26].

Microbial BVMOs have considerably broad substrate scope, for example the PockeMO can oxidize both a and d rings of the steroidal substrates [26], and this versatility is related to the structure of substrate binding pocket, able to accept bulky substrates in diverse configurations [26,27]. Such an idea of configurational flexibility of steroid substrates was proposed already in 1967 on the basis of diverse metabolic fates of steroids biotransformed by *Aspergillus tamarii* cultures [28]. Some recent examples of the presence of steroidal BVMOs in fungal biotransformation pathways are: lactonization of progesterone and 5-ene steroids [29,30]; lactonization of dehydroepiandrosterone (DHEA), pregnenolone, and androstenedione by filamentous fungi of genus *Penicillium* [31,32]; lactonization of DHEA by *Aspergillus parasiticus* [33]; ring-d lactonization of steroidal C-17 ketones to 11α-hydroxy derivatives by *Beauveria bassiana* [34]; activity of *Penicillium lanosocoeruleum* in ring-d lactonization of C_19_-steroids [35] and pregnene-based steroids [36]; biotransformation of DHEA into hydroxylated steroid lactones *Spicaria fumoso-rosea* [37]; diverse biotransformation routes of steroids, including ring-d lactonization, in the cultures of *Penicillium notatum* [24] and *Aspergillus terreus* [38]; formation of testololactone from diverse steroidal substrates with the use of a multifunctional strain of *Penicillium simplicissimum* [39]; cascade of DHEA biotransformations by *Beauveria* species [40]; and the formation of new derivatives of 3β-acetyloxy-5α-chloro-6,19-oxidoandrostan-17-one [41]. This rich background of BVMO activity studies in fungal species, combined with the outlined above need for further research in the field of biotransformations, prompted us to investigate in detail metabolic fates of DHEA (**1**), epiandrosterone (**2**), androsterone (**3**), androstenedione (**4**), 19-OH-androstenedione (**5**), testosterone (**6**), 19-nortestosterone (**7**), progesterone (**8**), and pregnenolone (**9**) in the cultures of *Penicillium vinaceum* AM110. The transformation progress was monitored by GC and TLC analysis and the resulting products were identified on the basis of chromatographic and spectral data.

## 2. Results and Discussion

**Scheme 1:**
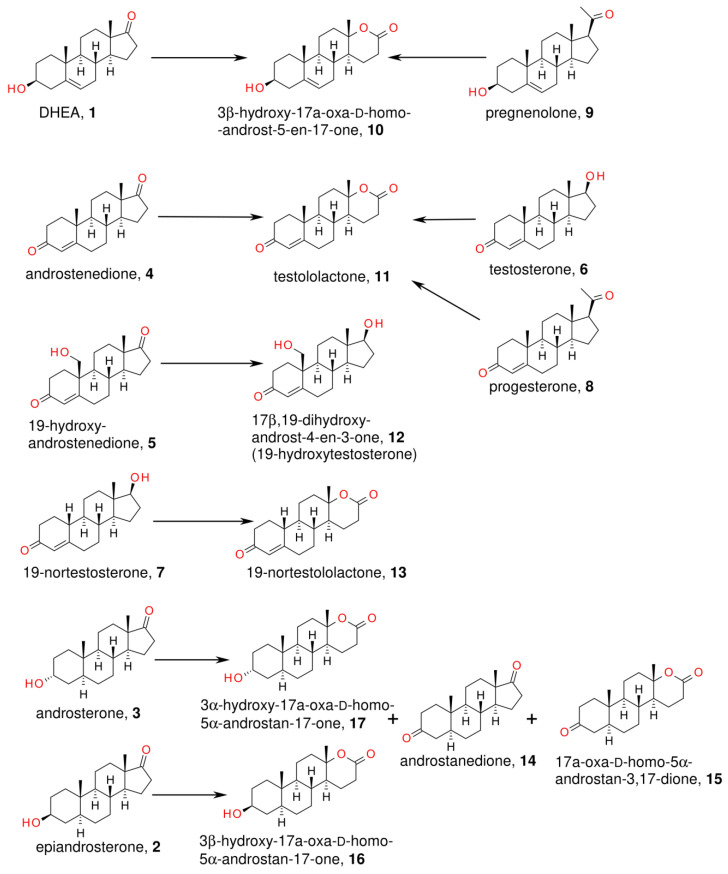
Biotransformation of steroidal substrates **1**–**9** by *P. vinaceum* AM110—overview of the substrate and product structures.

Biotransformations of the chosen substrates by *Penicillium vinaceum* AM110 proceed according to one of two distinct schemes. Substrates **1** and **4**–**9** undergo Baeyer-Villiger lactonization in ring d, and no other detectable products are formed. On the other hand, transformation of compounds **2** and **3**, the only 5α-saturated steroids in the investigated series of substrates, leads to a mixture of products. For this reason we have carried out transformations of **2** and **3** with time course monitoring of the composition of the reaction mixture. These two schemes of biotransformation will be described in separate subsections below. Scheme 1 presents the structures of the investigated compounds: substrates **1**–**9** and the corresponding products.

### 2.1. Baeyer-Villiger Oxidation of Steroidal Compounds by P. vinaceum AM110

Our earlier studies with biotransformations of steroids by the fungi of genus *Penicillium* [31,32,35,36] have revealed large diversity of metabolic fates of the substrates. Baeyer-Villiger oxidation, isomerizations, and hydroxylations with diverse product stereochemistry were found. On the other hand, the biocatalyst used in the current study, *Penicillium vinaceum* AM110, prefers to carry out only the BVO process for most of the substrates: **1**, **4**, and **6**–**9** (see Scheme 1 for a structural overview, and Table 1 for the detailed results of the transformations; in some cases the products were compounds for which we had no standard samples to compare with using the GC and TLC experiments, and these compounds were identified on the basis of NMR spectroscopy—see Section 2.3). In most cases the conversion ratio exceeds 90% in two days. An interesting exception is provided by 19-hydroxyandrostenedione (**5**) where, after 10 days of transformation, only half of the substrate was transformed. The parent compound of **5**, androstenedione (**4**), is converted in 95% into testololactone in 48 h. The ring-d Baeyer-Villiger lactonization of **4** was also the only process for this substrate in the cultures of *P. lilacinum* [31] and *P. camemberti* [32]. Its 19-OH derivative **5** is not affected by the BVMO, but it undergoes reduction at the same position C-17. Thus, introduction of the 19-OH function seems to obstruct the action of the fungal BVMO but not the action of a steroid 17β-HSD (17β-hydroxysteroid dehydrogenase).

An interesting perspective on the inhibition of the BVMO by the presence of the 19-OH function is provided by another pair of substrates, testosterone (**6**) and 19-nortestosterone (**7**). In this case, the 19-methyl group present in **6** is replaced by a hydrogen atom in **7**. This has only a moderate effect on the transformation kinetics: **6** is totally converted into testololactone in two days, while **7** is still present in minute amount (2%) after six days of transformation. However, both **6** and **7** are converted into the corresponding lactones, and no traces of any reduced derivatives were observed. In the presented studies, in the course of a six-day biotransformation 19-nortestololactone (**13**) was obtained with good yield of 90%. An established biosynthetic route to 19-nortestololactone utilizes 72 h transformation of 19-nortestosterone by *Aspergillus tamarii* with 70% yield (16 × 50 mg/500 mL) [23]. In another known example of transformation of 19-nortestosterone by *Penicillium notatum*, 19-nortestololactone was obtained with 93% yield, but at lower substrate concentration (100 mg/300 mL medium) [24] than in the current report. As a result of the 10-day transformation of 19-hydroxyandrostenedione (**5**), 17β,19-dihydroxyandrost-4-en-3-one (**12**) (19-hydroxytestosterone) was obtained with 46% yield. In the case of **5**, the BVMO activity and formation of the ring-d lactone derivative were not observed. To the best of our knowledge, we present the first described case of microbiological formation of 19-hydroxytestosterone from 17β,19-dihydroxyandrost-4-en-3-one in the culture of *P. vinaceum* (obtained chemically by Ehrenstein and Otto [42]).

These results confirm the preference of many steroid substrates to undergo lactonization, observed in many studies [29,30,31,32,37]. This preference for lactonization includes also the fact that many of the substrates studied here are not lactones, but 17-hydroxy, 17-acetyl etc. derivatives. However, the compounds differing only by the C-17 function are converted to the same product: **1** as well as **9** yield 3β-hydroxy-17a-oxa-d-homo-androst-5-en-17-one, while **4**, **6,** and **8** yield testololactone. Thus, successful BVMO activity requires also effective preceding steps leading to the cleavage or oxidation of the substituent at C-17, which was noted already by Sebek et al. [43].

The performance of BVMOs of the *Penicillium vinaceum* AM110 strain is comparable to other species of the genus *Penicillium*. For example, *P. lanosocoeruleum* converts progesterone to testololactone in 88% yield in 72 h (the value very similar to the current study), but pregnenolone is converted into a mixture of products, with only 44% yield of testololactone in 72 h [36] (in the current study on *P. vinaceum*, testololactone is the sole detected product with 91% yield after 48 h). *P. lanosocoeruleum* converts DHEA (**1**) solely to testololactone with 96% yield in 24 h [35], which is a different route, including isomerization, than in the current study, where only BVO is observed. Interestingly, other *Penicillium* species also differ in their ability of DHEA conversion: *P. lilacinum* AM111 transforms DHEA completely in 30 h into the same product as in the current study, 3β-hydroxy-17a-oxa-d-homo-androst-5-en-17-one [31], while *P. camemberti* AM83 exhibits two distinct metabolic routes for DHEA, both however converging into testololactone (85% yield after 24 h) [32].

Summarizing the ability of *P. vinaceum* to transform androstene substrates, we note that the metabolic fate of these substrates, leading to the corresponding lactones, is rather less complicated than in the other species of *Penicillium* genus, and the transformation kinetics and achieved yields are very promising.

### 2.2. Transformations of 5α-Saturated Steroids in the Cultures of P. vinaceum AM110

**Figure 1:**
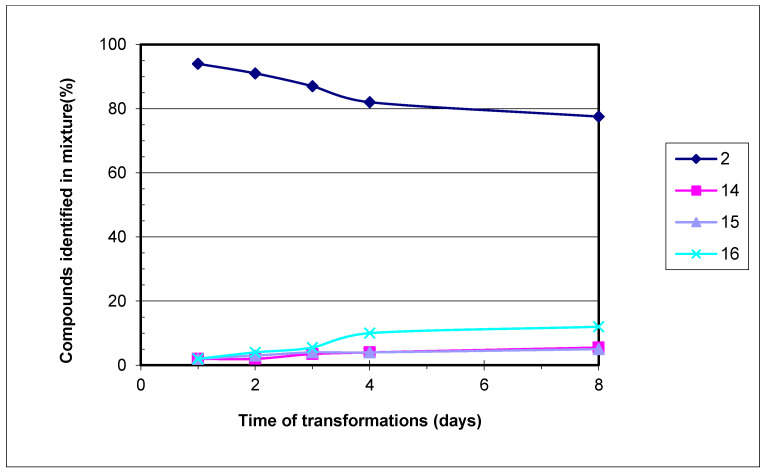
The time course of the transformation of epiandrosterone (**2**) by *Penicillium vinaceum.*

**Figure 2:**
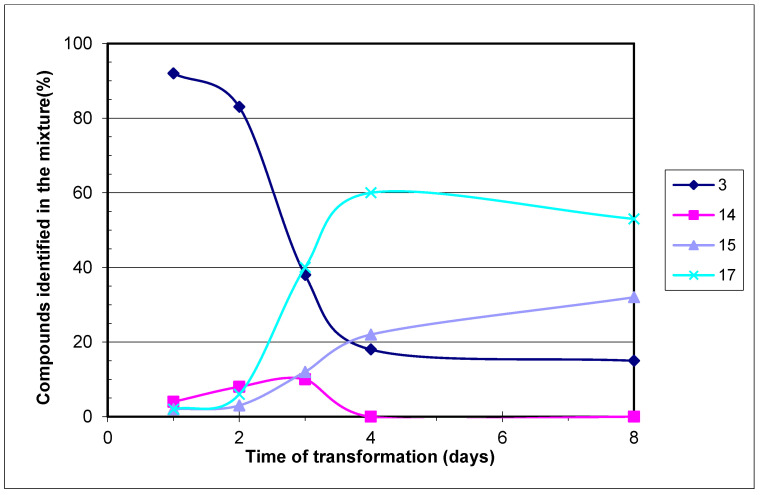
The time course of the transformation of androsterone (**3**) by *Penicillium vinaceum.*

The metabolic fate of androsterone (**3**) and epiandrosterone (**2**), representative biologically active 5α-saturated steroids, turns out to be more complicated than the transformation of the unsaturated steroids described above. Initial tests indicated that *P. vinaceum* is able to transform **2** and **3** into a mixture of products oxidized at C-17 (BVMO activity, products **16** and **17**), at C-3 (product **14**, oxidation of the hydroxyl group to the corresponding ketone) or at both C-3 and C-17 (product **15**). See Scheme 1 for the structures of the substrates and products. This fact, already observed, e.g., for *P. lanosocoeruleum* [35], prompted us to carry out transformations aimed at the time course analysis of the transformation progress. The results are summarized in Table 2, Figure 1 and Figure 2.

An interesting feature of the *P. vinaceum* enzymatic system is strong discrimination of the oxidation kinetics on the basis of the C-3 stereochemistry. While **2**, a 3β-hydroxy steroid, is not readily transformed (even after eight days of transformation, still 77.5% of the unreacted **2** is present in the reaction mixture), its 3α-hydroxy stereoisomer **3** is converted in 85%, and the transformation is especially fast on the third day of the experiment. The oxidation kinetics is, however, generally noticeably slower than for the 5-ene steroids described in Section 2.1.

While transformation of epiandrosterone (**2**) progresses slowly and concentrations of products grow monotonically, the case of androsterone (**3**) is more interesting. Androstanedione, the carbonyl derivative of **3**, formed by oxidation of the hydroxyl group at C-3, undergoes between 72 and 96 h further conversion, most probably BVO to the lactone **15**. This assumption is supported by the fate of 3α-hydroxy-17a-oxa-d-homo-5α-androstan-17-one (**17**), the product of the BVO retaining the 3α-hydroxy function. This compound is the predominant one within the experimental time scale, but its amount decreases in the final period of transformation, while new, corresponding amount of **15** is formed. This progression of product concentrations shows that the enzyme oxidizing the hydroxyl group at C-3 to the ketone acts slower than the BVMO. Changing the configuration from 3β-hydroxy in epiandrosterone (**2**) to 3α-hydroxy in androsterone (**3**) slowed down the transformation process and activity of Baeyer–Villiger monooxygenase.

It is also interesting to compare the transformation of DHEA (**1**) and its 5α-saturated analog—epiandrosterone (**2**). The rapid conversion of **1** to 3β-hydroxy lactone (**10**) with high yield indicates that the substrate is the inducer of BVMO. In the case of transformation of **2**, we observed a significant reduction in the transformation rate and the catalytic activity of BVMO, indicating that epiandrosterone (**2**) may be the BVMO inhibitor.

### 2.3. Spectroscopic Identification of Products **12** and **13**

The structures of the obtained and isolated products **12** and **13** were established by using spectroscopic techniques (IR, ^1^H-NMR and ^13^C-NMR). The assumed structures were confirmed by comparison of the characteristic shift values of selected, diagnostic signals of the products and starting compounds.

The ^1^H NMR spectrum of **12** features a characteristic signal at δ_H_ 3.63 ppm (t, *J* = 8.5 Hz, 17α-H) from the proton at C-17. Further, in the ^13^C NMR spectrum of **12** a new signal appears at δ_C_ 81.5 ppm and no signal at δ_C_ 220.3 ppm is observed (present in the substrate), which unanimously indicates reduction of the ring-d carbonyl group to the hydroxyl group at 17β position, accompanied by an upfield shift of the C-18 methyl group signal (Δ0.122 ppm) and downfield shift of the 4-H signal (Δ0.013 ppm) in the ^1^H NMR spectrum. The signals at δ_H_ 3.90 ppm (d, *J* = 11 Hz) and at δ_H_ 4.06 ppm (dd, *J_1_* = 1 Hz, *J_2_* = 11 Hz) are formed by two protons of the methylene group at C-19, and indicate the close proximity of a hydroxyl group proton (19-CH_2_-OH). The signal appearing as a broad singlet at δ_H_ 5.94 ppm points to the presence of a double bond at the C-4 carbon atom.

The analysis of the ^1^H NMR and ^13^C NMR spectral data indicates that the formed product **12** is 17β,19-dihydroxyandrost-4-en-3-one.

In the ^1^H NMR spectrum of **13** a significant downfield shift was observed for the 18-methyl resonance signals in comparison with the substrate (from δ_H_ 0.81 to δ_H_ 1.36 ppm). It was consistent with an oxygen atom insertion into the ring d. This was supported by the ^13^C NMR spectrum in which the C-13 resonance signal had undergone a downfield shift of ca. 52 ppm (from δ_C_ 30.7 to δ_C_ 82.9 ppm). The lactonization via Baeyer–Villiger oxidation in the ring d of 19-nortestololactone was confirmed by the appearance of the signal at δ_C_ ca. 171 ppm (C-17). The H-17α triplet characteristic for 19-nortestosterone (δ_H_ 3.66 ppm, *J* = 8.5 Hz) has disappeared, while the remaining signal of olefinic proton δ_H_ ca. 5.84 ppm in the ^1^H NMR spectrum and a non-protonated signal at δ_C_ 199.5 ppm in the ^13^C NMR spectrum indicate the presence of 3-oxo-4-en moiety in the obtained product. The band at 1725 cm^−1^ in the IR spectrum confirmed the formation of the δ-lactone structure. These facts are consistent with identification of **13** as 19-nortestololactone.

## 3. Materials and Methods

### 3.1. Chemicals and Microorganism

The substrates, namely DHEA (dehydroepiandrosterone, 3β-hydroxyandrost-5-en-17-one, **1**), epiandrosterone (3β-hydroxy-5α-androstan-17-one, **2**), androsterone (3α-hydroxy-5α-androstan-17-one, **3**), androstenedione (androst-4-ene-3-one, **4**), 19-hydroxyandrostenedione (19-hydroxyandrost-4-ene-3,17-dione, **5**), testosterone (**6**), 19-nortestosterone (**7**), progesterone (**8**), and pregnenolone (**9**) standards, were purchased from Sigma-Aldrich Chemical Co. (St. Louis, MO, USA)

The fungal strain *Penicillium vinaceum* AM110 used for biotransformations was taken from the collection of Department of Chemistry, Wrocław University of Environmental and Life Sciences, Wrocław, Poland. The strain was maintained on Sabouraud 4% dextrose-agar slopes and freshly subcultured before use in the transformation experiments.

### 3.2. General Conditions of Cultivation and Biotransformation

General experimental and fermentation details were described previously [31]. After 4 days of growth period of *P. vinaceum* AM110, a given substrate was added to each flask in an amount necessary to obtain 0.30 g L^−1^ final substrate concentration (0.50 g L^−1^ final substrate concentration for 19-nortestosterone). The transformation was carried out at 25 °C in a rotary shaker (180 rpm) for eight days (for 19-hydroxyandrostenedione the time was prolonged to 10 days, while for 19-nortestosterone, the transformation lasted six days) with periodic checks of the presence of the substrate in the reaction mixture and the stability of the obtained products (see below for the details of the checks). Each experiment was performed with three replications (five replications for transformation of 19-hydroxyandrostenedione).

The monitoring of the time-dependent progress of the bioconversion and determination of the metabolic pathways of substrates was carried out as follows. The 5-mL samples of the broth were taken out at regular intervals (24, 48, 72, and 96 h from the start) from the reaction flask, extracted with organic solvents (chloroform or methylene chloride), and analyzed by comparison of the GC and TLC data with those of authentic samples. The final samples, taken at the end of transformation (6, 8, or 10 days, depending on the substrate, as described above), served as the control of the extended metabolic fate of the substrates and products.

### 3.3. Isolation and Identification of the Products

Briefly, 3α-Hydroxy-17a-oxa-d-homo-5α-androstan-17-one (**17**), 3β-hydroxy-17a-oxa-d-homo-5α-androstan-17-one (**16**) and 17a-oxa-d-homo-5α-androstan-3,17-dione (**15**) were obtained previously in our laboratory by transformation by *Penicillium lanosocoeruleum* KCH 3012 as described in our article [35]. 3β-Hydroxy-17a-oxa-d-homo-androst-5-en-17-one (**10**) and testololactone (**11**) were obtained previously in our laboratory by transformation of DHEA by *Penicillium lilacinum* AM111 [31]. Androstanedione (5α-androstan-3,17-dione) (**14**) was obtained by the oxidation of androsterone (**3**) with Jones’ reagent [35]. The aforementioned compounds were used as analytical standards for the time course experiments.

Identification of the transformation products of substrates **1**–**9** was carried out on the basis of the GC and TLC (for **1**: hexane/acetone (2:1 *v:v*); for **2**: hexane/acetone (1:1 *v:v*); for **3**: hexane/acetone (2:1 *v:v*); for **4**: hexane/acetone (2:1 *v:v*), for **6**: methyl chloride/acetone (4:1 *v:v*), for **8** ethyl acetate/methyl chloride/acetone (3:1:1 *v:v:v*), for **9**: ethyl acetate/methyl chloride/acetone (3:1:1 *v:v:v*)) analysis by comparison of the retention times (*Rt*) with the *Rt* values of the standards available in our laboratory, which have been already identified in our earlier studies [31,35].

Isolation and identification (by ^1^H NMR and ^13^C NMR) of the products were carried out for 19-hydroxyandrostenedione (**5**) and 19-nortestosterone (**7**), the two substrates purchased from Sigma-Aldrich Poland company, Poznań, Poland.

The products of biotransformation were extracted three times with chloroform (for substrates **1**–**4**, **6**–**9**) or chloride methylene (for substrate **5**). The organic extracts were dried over anhydrous magnesium sulfate, concentrated in vacuo, and analyzed by TLC and GC. Products were separated by column chromatography on silica gel (Silica gel 60, 63–200 µm, 70–230 mesh, Sigma-Aldrich, Buchs, Switzerland) with hexane/acetone (2:3 *v:v*) for 19-hydroxyandrostenedione, and hexane/acetone (1:1 *v:v*) for 19-nortestosterone, as eluents. TLC was carried out with Kieselgel 60 F_254_ plates (Merck, Darmstadt, Germany) using the same eluents. In order to develop the image, the plates were sprayed with solution of methanol in concentrated sulfuric acid (1:1) and heated to 120 °C for 3 min. GC analysis was performed using Hewlett Packard 5890A Series II GC instrument (FID, carrier gas H_2_ at flow rate of 2 mL min^−1^, Hewlett-Packard Company, Wilmington, DE, USA) with a DB-5MS column cross-linked phenyl-methylsiloxane, 30 m × 0.32 mm × 0.25 μm film thickness. The following program was used in the GC analysis: 220 °C/1 min, gradient 4 °C/min to 270 °C and 30 °C/min to 300 °C/3 min (for **1**–**6**, **8**, **9** substrates); 220 °C/1 min, gradient 4 °C/min to 260 °C and 30 °C/min to 300 °C/3 min (for **7**); injector and detector temperatures were 300 °C. The NMR spectra (for obtained products of transformation of 19-hydroxyandrostenedione and 19-nortestosterone) were measured in CDCl_3_ and recorded on a DRX 600 MHz Bruker Avance spectrometer (Bruker Polska, Poznań, Poland). Characteristic ^1^H- and ^13^C-NMR shift values in comparison to the starting compounds were used to determine structures of metabolites, and in combination with DEPT analysis to identify the nature of the carbon atoms. Elemental analysis was performed on vario EL III analyzer (Elementar Analysensysteme GmbH, Hanau, Germany). Infrared spectra (IR) were recorded in KBr disc on a Mattson IR 300 Spectrometer (Mattson-Garvin Company, Maitland, FL, USA). Melting points were determined on a Boetius melting point apparatus and are uncorrected.

### 3.4. Spectral Data of the Isolated Transformation Products

*17β,19-dihydroxyandrost-4-en-3-one* (**12**) (60 mg, 40% mol): C_19_H_28_O_3_, Found: C, 74.89; H, 9.26. Requires C, 74.96; H, 9.27. m.p. 200–202 °C (lit. 200-204 °C [42]). IR: υ_max_ (cm^−1^): 3410, 1660, 1615 in good agreement with [42].

^1^H NMR (CDCl_3,_ 600 MHz,) δ_H_: 0.77 (3H, s, 18-H), 3.63 (1H, t, *J* = 8.5 Hz, 17α-H), 3.90 (1H, d, *J* = 11 Hz, 19-CH_2_-), 4.06 (1H, dd, *J_1_* = 1 Hz, *J_2_* = 11 Hz, 19-CH_2_-), 5.94 (1H, br s, 4-H); ^13^C NMR (CDCl_3_, 151 MHz) δ_C_: 11.1 (C-18), 21.2 (C-11), 23.2 (C-15), 30.4 (C-16), 31.7 (C-7), 33.4 (C-6), 33.5 (C-2), 35.0 (C-8), 36.3 (C-12), 36.7 (C-1), 42.9 (C-13), 43.9 (C-10), 50.8 (C-14), 54.0 (C-9), 66.0 (C-19), 81.5 (C-17), 127.0 (C-4), 166.6 (C-5), 199.9 (C-3). The NMR spectra of **12** are attached as Supplementary Figures S1 and S2.

*19-Nortestololactone* (**13**) (127 mg, 85% mol): C_18_H_24_O_3_, Found: C, 74.90; H, 8.36. Requires C, 74.97; H, 8.39; m.p. 195–198 °C (lit. 196–199 °C [23]), IR: υ_max_ (cm^−1^): 1725, 1670, 1610.

^1^H NMR (CDCl_3_, 600 MHz,) δ_H_: 1.36 (3H, s, 18-H), 5.84 (1H, s, 4-H); ^13^C NMR (CDCl_3_,151 MHz): 19.8 (C-18), 20.0 (C-15), 26.3 (C-1), 27.0 (C-11), 28.5 (C-16), 29.7 (C-7), 35.0 (C-6), 36.4 (C-2), 38.9 (C-8), 42.3 (C-10), 42.6 (C-12), 45.03 (C-14), 48.2 (C-9), 82.9 (C-13), 124.8 (C-4), 164.7 (C-5), 171.2 (C-17), 199.5 (C-3). The spectroscopic data are in agreement with those reported in the literature [24]. The NMR spectra of **13** are attached as Supplementary Figures S3 and S4.

## 4. Conclusions

Biotransformations of nine steroidal substrates in the cultures of the filamentous fungus *Penicillium vinaceum* AM110 have been carried out. The series of androstene substrates underwent mostly Baeyer–Villiger oxidation (lactonization), without side processes; the metabolic fate of these substrates is therefore less complicated than in the other species of *Penicillium* genus, while the transformation kinetics and achieved yields are very promising. Interesting inhibition of the BVMO activity was observed when a hydroxyl function was inserted at the C-19 methyl group. Instead of any oxidation process, the C-17 carbonyl group of 19-hydroxyandrostenedione was reduced to the hydroxyl group.

The fate of androstane steroids, epiandrosterone and androsterone, was more complicated. Two possible sites of oxidation, the C-3 hydroxyl and the C-17 carbonyl groups, were oxidized correspondingly to the ketone and to the δ-lactone with different rates. This allows for the potential design of the biotransformation process to obtain preferentially only one of the oxidation products. The transformation of epiandrosterone in comparison with its unsaturated analogue (DHEA) and androsterone was much slower and occurred with much lower efficiency.

The presented results suggest usefulness of the *Penicillium vinaceum* AM110 strain in the commercial preparation of biologically active δ-lactones of androstene and androstane series via microbial Baeyer–Villiger oxidation.

## Figures and Tables

**Table 1:** The final outcome of the transformation of steroidal compounds by *Penicillium vinaceum*.

Substrate	R_t_ (min)	Time (h/d)	Steroidal Compounds Identified in the Mixture (%) ^a^	Yield (%) ^a,c^
DHEA (**1**)	4.20	24 h	DHEA	-
	7.79		3β-Hydroxy-17a-oxa-d-homo-androst-5-en-17-one ^b^ (**10**)	96
Androstenedione (**4**)	5.29	48 h	Androstenedione	5
	9.32		Testololactone ^b^ (**11**)	95
19-Hydroxyandrostenedione (**5**)	9.26	10 d	19-Hydroxyandrostenedione	54
	9.43		17β,19-dihydroxyandrost-4-en-3-one (**12**)	46 (40)
Testosterone (**6**)	5.61	48 h	Testosterone	-
	9.32		Testololactone ^b^ (**11**)	100
19-Nortestosterone (**7**)	5.19	6 d	19-nortestosterone	2
	9.09		19-nortestololactone (**13**)	90 (85)
Progesterone (**8**)	7.51	72 h	Progesterone	13
	9.32		Testololactone ^b^ (**11**)	87
Pregnenolone (**9**)	6.22	48 h	Pregnenolone	9
	7.79		3β-Hydroxy-17a-oxa-d-homo-androst-5-en-17-one ^b^ (**10**)	91

^a^ Determined by GC analysis of the crude chloroform (chloride methylene for (**5**)) extracts. ^b^ Identified in GC and TLC on the basis of comparison with standard samples. ^c^ Isolated yield in parentheses.

**Table 2:** The time course of the transformation of epiandrosterone and androsterone by *Penicillium vinaceum.*

Substrate	*R_t_* (min)	Compounds Identified in the Mixture (%) ^a^	Time of Transformation (h)				
			24	48	72	96	8 d
Epiandrosterone (**2**)	4.51	Epiandrosterone	94	91	87	82	77.5
	4.98	Androstanedione ^b^ (**14**)	2	2	3.5	4	5.5
	8.95	17a-Oxa-d-homo-5α-androstan-3,17-dione ^b^ (**15**)	2	3	4	4	5
	8.43	3β-Hydroxy-17a-oxa-d-homo-5α-androstan-17-one ^b^ (**16**)	2	4	5.5	10	12
Androsterone (**3**)	4.68	Androsterone	92	83	38	18	15
	4.98	Androstanedione ^b^ (**14**)	4	8	10	-	-
	8.95	17a-Oxa-d-homo-5α-androstan-3,17-dione ^b^ (**15**)	2	3	12	22	32
	8.48	3α-Hydroxy-17a-oxa-d-homo-5α-androstan-17-one ^b^ (**17**)	2	6	40	60	53

^a^ Determined by GC analysis of the crude chloroform extracts. ^b^ Identified in GC and TLC on the basis of comparison with standard samples.

## Supplementary Materials

The following is available online, a PDF file containing Figures S1–S6: ^1^H and ^13^C NMR spectra of products **12** and **13**, GC spectra of products **12** and **13** obtained during time course experiments.
