# Supplementary material for: Microbial Modifications of Androstane and Androstene Steroids by Penicillium vinaceum
Source: Molecules. 2020 Sep 15;25(18):4226. doi: 10.3390/molecules25184226 (PMC7570940; doi:10.3390/molecules25184226)
Supplement: Supplementary file 1 [file molecules-25-04226-s001.pdf]

Supporting Information

Microbial modifications of androstane and androstene steroids by *Penicillium vinaceum*

Anna Panek *, Paulina Łyczko, and Alina Świzdor

* Correspondence: anna.panek@upwr.edu.pl

Department of Chemistry, Wrocław University of Environmental and Life Sciences, Norwida 25, 50-375 Wrocław, Poland

Contents:

Figure S1. ^{1}H NMR spectrum of 17β,19-dihydroxyandrost-4-en-3-one (**12**) 2

Figure S2. ^{13}C NMR spectrum of 17β,19-dihydroxyandrost-4-en-3-one (**12**) 3

Figure S3. ^{1}H NMR spectrum of 19-nortestololactone (**13**) 4

Figure S4. ^{13}C NMR spectrum of 19-nortestololactone (**13**) 5

Figure S5. GC spectrum of 17β,19-dihydroxyandrost-4-en-3-one (**12**) 6

Figure S6. GC spectrum of 19-nortestololactone (**13**) 7

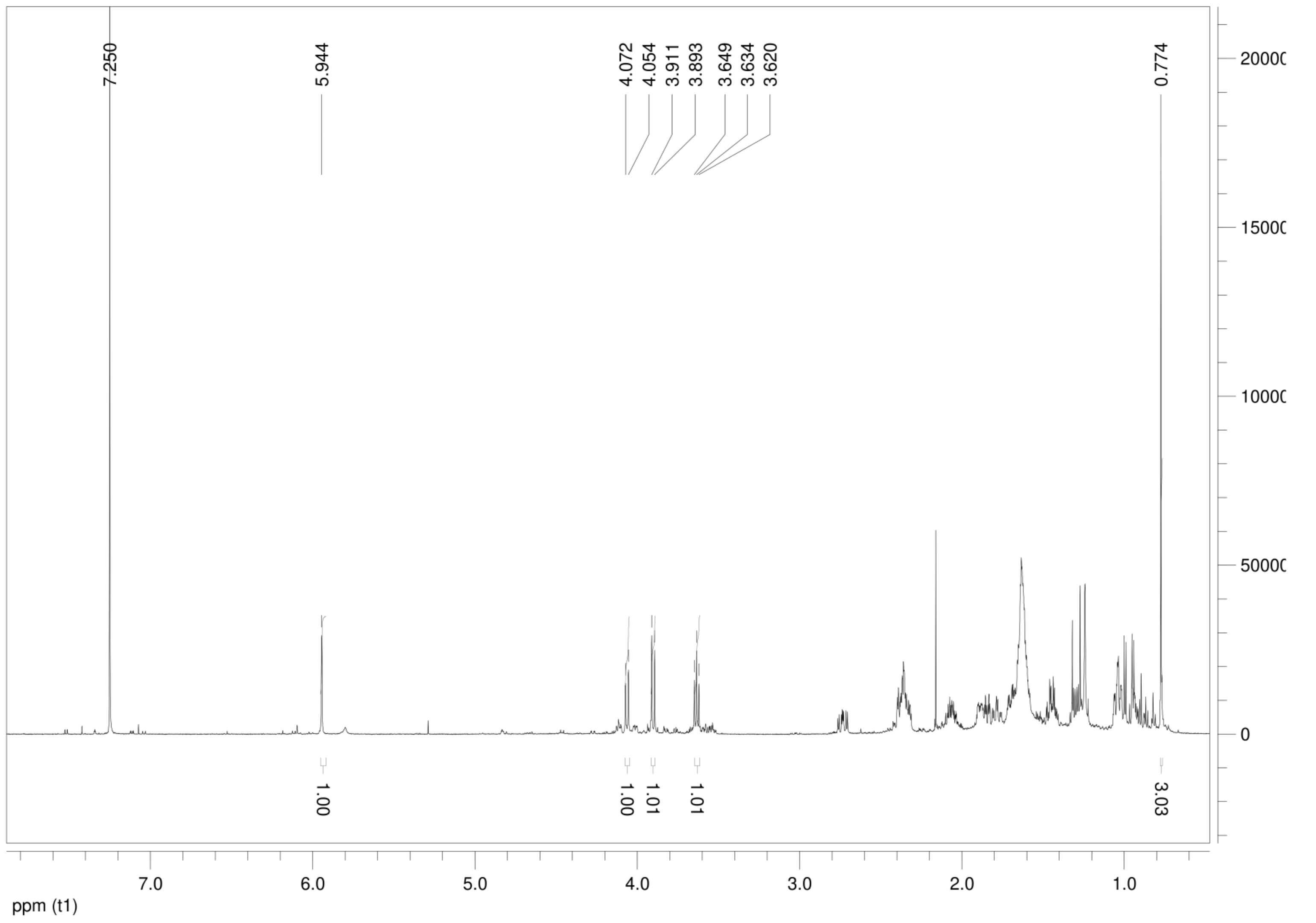

Figure S1.^{1}H NMR spectrum of 17β,19-dihydroxyandrost-4-en-3-one (**12**)

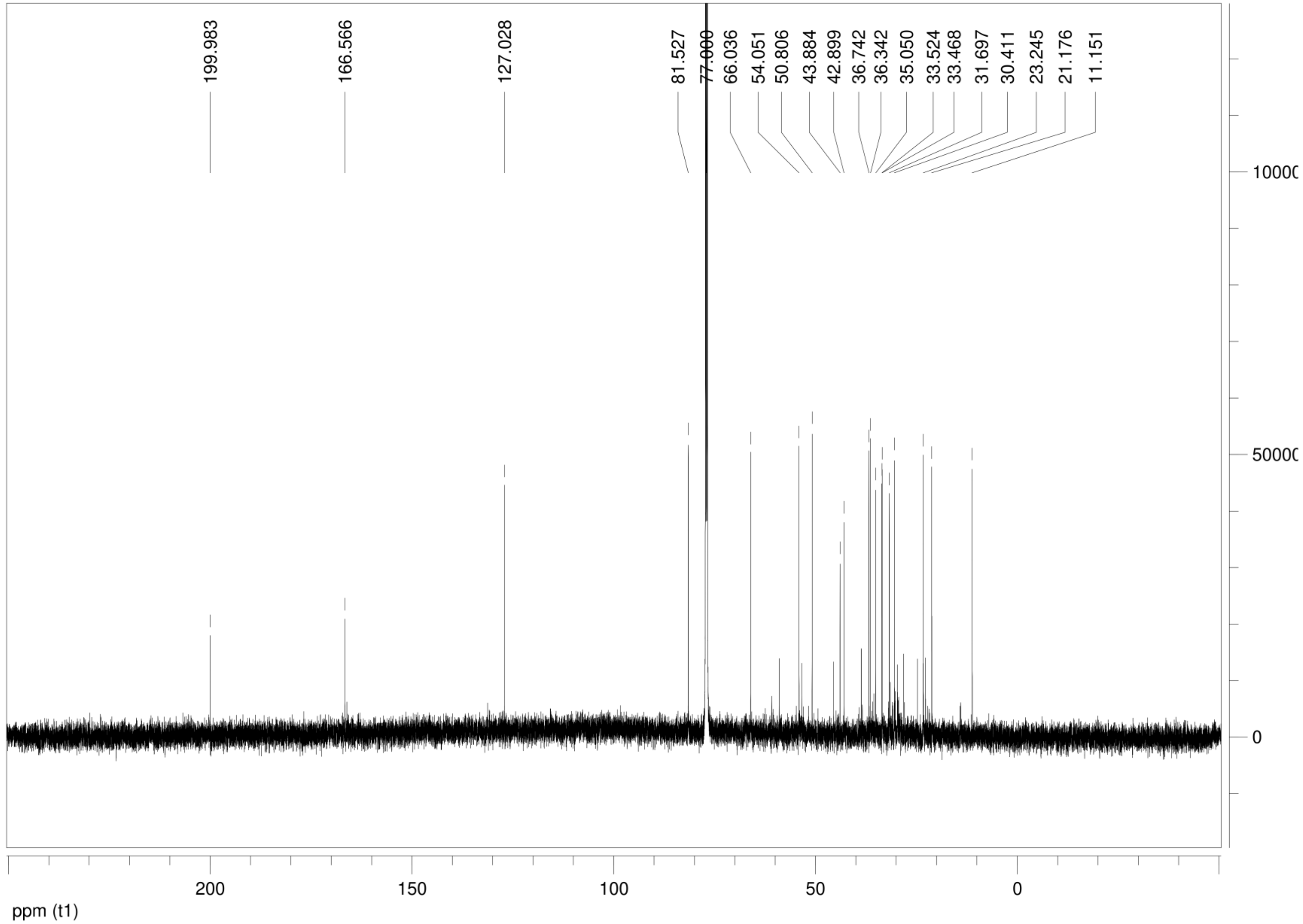

Figure S2. ^{13}C NMR spectrum of 17β,19-dihydroxyandrost-4-en-3-one (**12**)

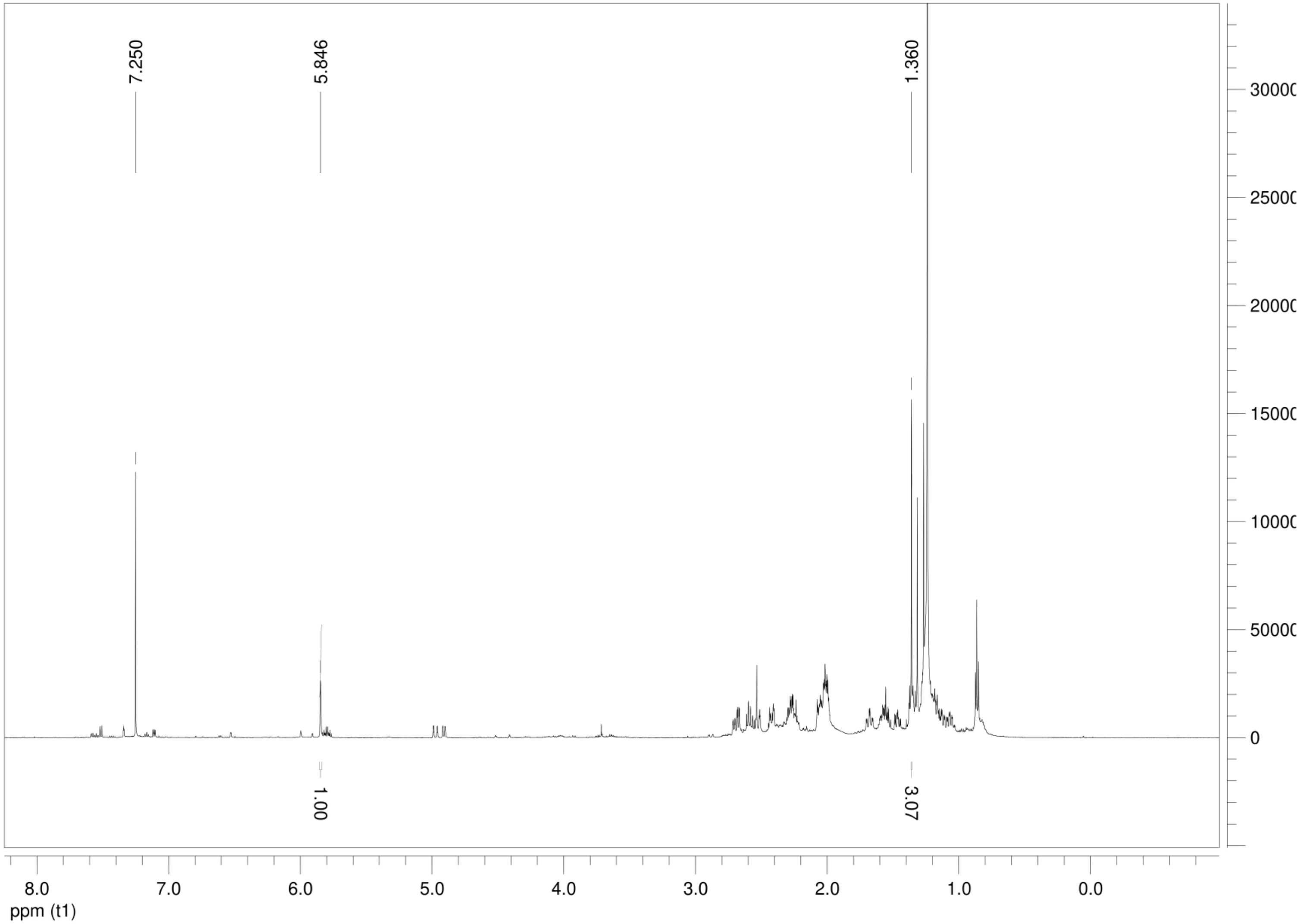

Figure S3.^{1}H NMR spectrum of 19-nortestololactone (**13**)

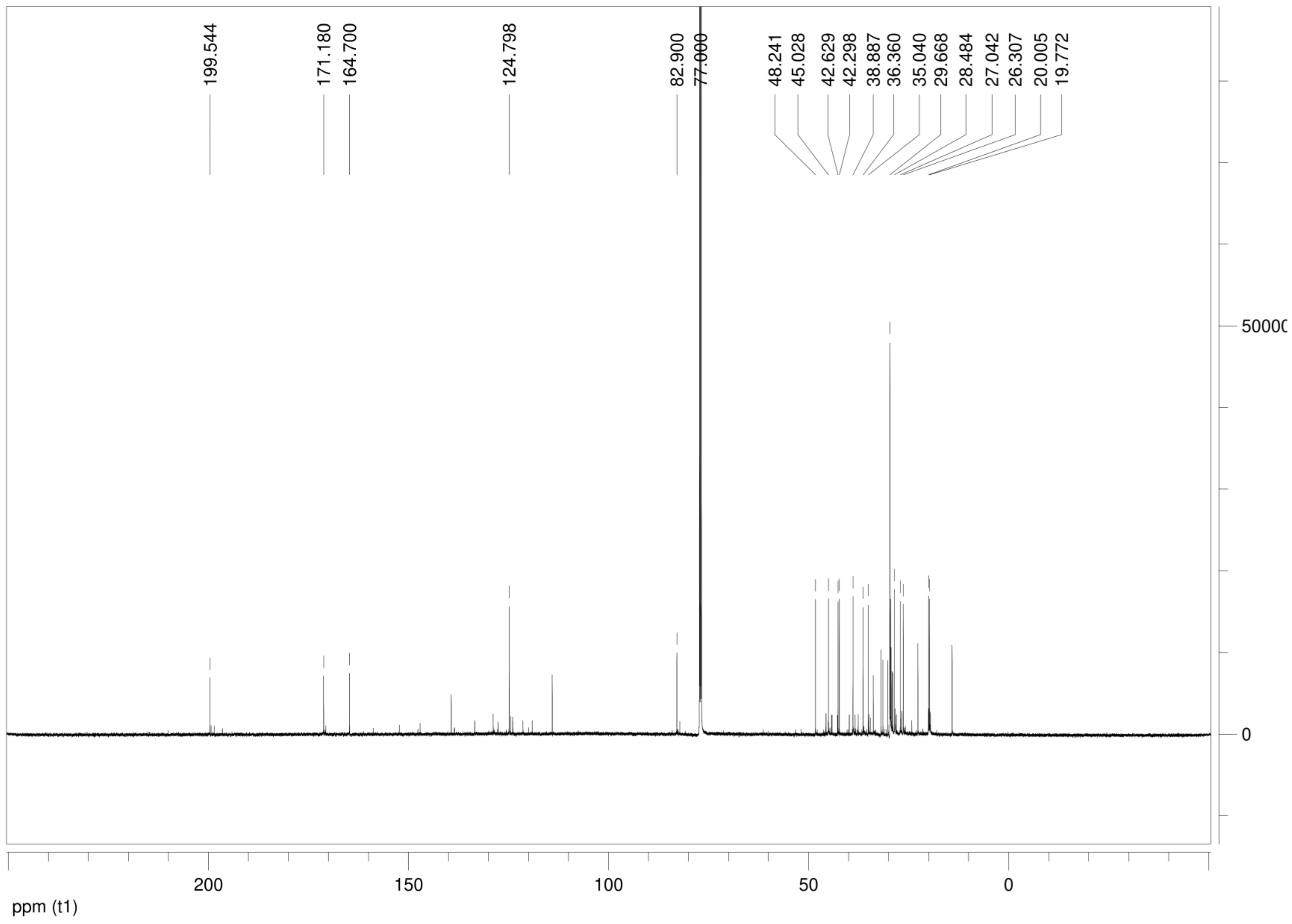

Figure S4.^{13}C NMR spectrum of 19-nortestololactone (**13**)

Additional Info : Peak(s) manually integrated

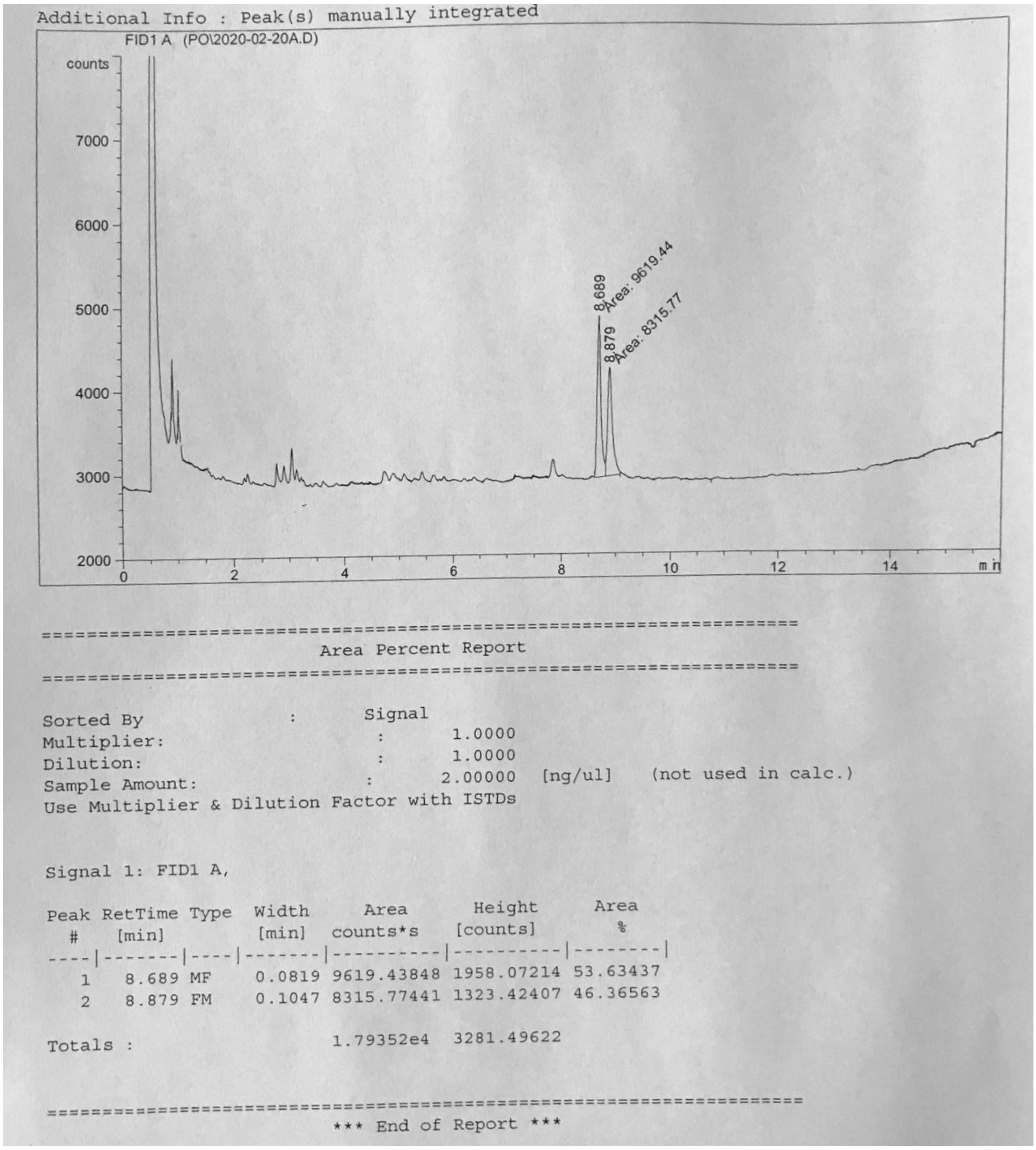

==

Area Percent Report

==

Sorted By : Signal
Multiplier: : 1.0000
Dilution: : 1.0000
Sample Amount: : 2.00000 [ng/ul] (not used in calc.)
Use Multiplier & Dilution Factor with ISTDs

Signal 1: FID1 A,

Peak #	RetTime [min]	Type	Width [min]	Area counts*s	Height [counts]	Area %
1	8.689	MF	0.0819	9619.43848	1958.07214	53.63437
2	8.879	FM	0.1047	8315.77441	1323.42407	46.36563
Totals :				1.79352e4	3281.49622	

==

*** End of Report ***

Figure S5. GC spectrum of 17β,19-dihydroxyandrost-4-en-3-one (**12**). This spectrum was obtained from extracts isolated during time course experiments and contains signals from the substrate **5** and the product **12**.

[illegible]

Additional Info : Peak(s) manually integrated

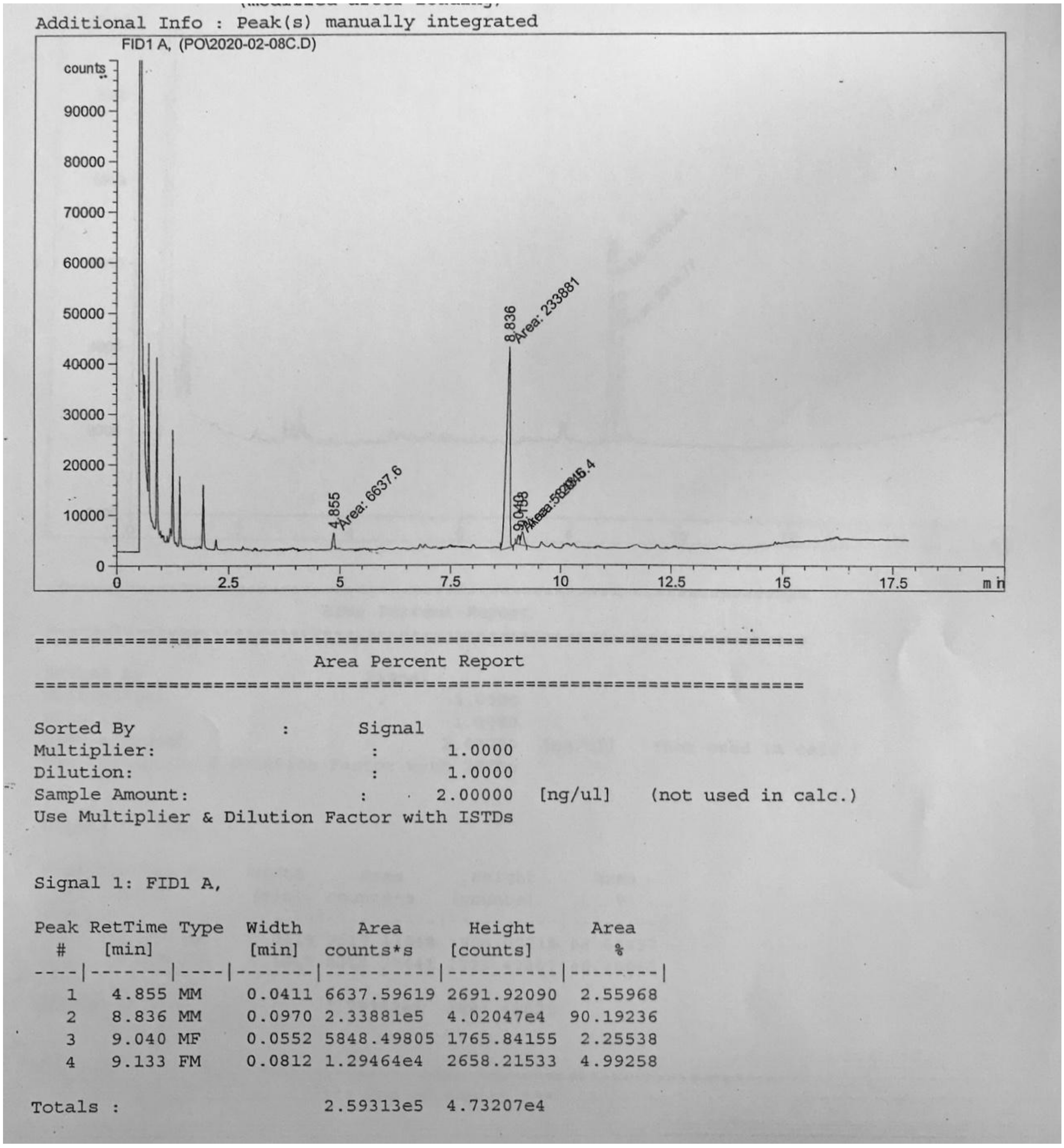

==

Area Percent Report

==

Sorted By : Signal
Multiplier: : 1.0000
Dilution: : 1.0000
Sample Amount: : 2.00000 [ng/ul] (not used in calc.)
Use Multiplier & Dilution Factor with ISTDs

Signal 1: FID1 A,

Peak #	RetTime [min]	Type	Width [min]	Area counts*s	Height [counts]	Area %
1	4.855	MM	0.0411	6637.59619	2691.92090	2.55968
2	8.836	MM	0.0970	2.33881e5	4.02047e4	90.19236
3	9.040	MF	0.0552	5848.49805	1765.84155	2.25538
4	9.133	FM	0.0812	1.29464e4	2658.21533	4.99258
Totals :				2.59313e5	4.73207e4	

Figure S6. GC spectrum of 19-nortestololactone (**13**). This spectrum was obtained from extracts isolated during time course experiments and contains signals from the substrate **7** and the product **13**.
